# Multiple paternity, fertilization success, and male quality: Mating system variation in the eelgrass, *Zostera marina*

**DOI:** 10.1002/ece3.11608

**Published:** 2024-06-25

**Authors:** Lauren R. Sgambelluri, Jessie C. Jarvis, Stephanie J. Kamel

**Affiliations:** ^1^ Department of Biology and Marine Biology, Center for Marine Science University of North Carolina Wilmington Wilmington North Carolina USA

**Keywords:** genetic diversity refuge, marine angiosperm, paternity skew, reproductive skew, seagrass, sexual reproduction

## Abstract

Genetic diversity can modulate a population's response to a changing environment and plays a critical role in its ecological function. While multiple processes act to maintain genetic diversity, sexual reproduction remains the primary driving force. Eelgrass (*Zostera marina*) is an important habitat‐forming species found in temperate coastal ecosystems across the globe. Recent increases in sea surface temperatures have resulted in shifts to a mixed‐annual life‐history strategy (i.e., displaying characteristics of both annual and perennial meadows) at its southern edge‐of‐range. Given that mating systems are intimately linked to standing levels of genetic variation, understanding the scope of sexual reproduction can illuminate the processes that shape genetic diversity. To characterize edge‐of‐range eelgrass mating systems, developing seeds on flowering *Z. marina* shoots were genotyped from three meadows in Topsail, North Carolina. In all meadows, levels of multiple mating were high, with shoots pollinated by an average of eight sires (range: 3–16). The number of fertilized seeds (i.e., reproductive success) varied significantly across sires (range: 1–25) and was positively correlated with both individual heterozygosity and self‐fertilization. Outcrossing rates were high (approx. 70%) and varied across spathes. No clones were detected, and kinship among sampled flowering shoots was low, supporting observed patterns of reproductive output. Given the role that genetic diversity plays in enhancing resistance to and resilience from ecological disturbance, disentangling the links between life history, sexual reproduction, and genetic variation will aid in informing the management and conservation of this key foundation species.

## Abstract 2

This study provides a thorough description of fine‐scale patterns in *Zostera marina*'s mating system at the species' southern edge‐of‐range, an area where eelgrass experiences frequent summer heat stress. Sampled populations displayed a reliance on sexual recruitment, and male quality was linked to reproductive success. Our findings serve as stepping stones to further understand the evolutionary processes underpinning seagrass genetic composition and life history, which is essential for informed conservation and management.

## 1 INTRODUCTION

Genetic diversity is a fundamental characteristic of populations, determining their response to a changing environment (Fisher, 1930) and playing a critical role in ecological function (Hughes et al., 2008; Whitham et al., 2006). Especially in systems where an organism serves as a foundation species, the functional traits displayed by different genotypes can have profound effects on community persistence and productivity (Reusch, 2006; Reynolds et al., 2012; Wimp et al., 2004) in ways comparable to species diversity in other systems (Crutsinger et al., 2006; Wimp et al., 2004). While multiple processes act to maintain genetic diversity, sexual reproduction remains the driving force, particularly over short time scales, and is highly dependent upon life‐history strategy and mating system (Williams, 1975).

Seagrasses (i.e., marine flowering plants) act as foundation organisms for highly productive nearshore habitats and provide essential ecosystem services such as habitat for an array of epifaunal species and fisheries, coastal protection from storm surge and wave action, sediment stabilization via root and rhizome growth, and the mitigation of excess nutrient loads and eutrophication (Duarte et al., 2013; Gillanders, 2006). Seagrass meadows are, however, experiencing a trajectory of decline, and a global net loss of 5602 km^2^ (19.1% of surveyed meadow area) has occurred since 1880 (Dunic et al., 2021). Rates of seagrass loss are comparable to those reported for mangroves, coral reefs, and tropical rainforests, which places seagrass meadows among the most threatened ecosystems on Earth (Waycott et al., 2009). On average, only 37% of restoration efforts are successful (Van Katwijk et al., 2016), highlighting the importance of conserving existing meadows before collapse occurs.

The seagrass *Zostera marina* (eelgrass) is commonly found in the temperate regions of the world (Green & Short, 2003) and experiences a range of abiotic and biotic stressors (Kendrick et al., 2019; Lefcheck et al., 2018; Orth et al., 2017). Such selective pressures can lead to changes in life‐history strategy and mating system dynamics (Cabaço & Santos, 2012). As such, *Z. marina*'s allocation to sexual reproduction varies across its geographic range (Phillips et al., 1983). Specifically, extreme environmental conditions result in annual populations characterized by yearly shoot mortality, development of only reproductive shoots, high seed production, and biennial establishment of seedlings (Phillips & Backman, 1983); within‐range, perennial populations are less reliant on flowering and sexual reproduction, additionally undergoing clonal extension of the rhizome to form ramets (Silberhorn et al., 1983). However, even in the same region, flowering can vary widely within and among populations for reasons still largely unknown (Von Staats et al., 2021). Perennial populations maintained by asexual, clonal growth were once assumed to be the primary life history strategy of *Z. marina* (Den Hartog, 1970). Now, sexual recruitment from seed is becoming more widely acknowledged as an important driver in both the maintenance and expansion of perennial (Johnson et al., 2020) and annual populations (Xu et al., 2018).

At its southern edge‐of‐range, *Z. marina* populations experience annual loss of biomass due to heat stress and are shifting primarily to a mixed‐annual life‐history strategy characterized by annual, complete loss of biomass, development of both vegetative and reproductive shoots, reestablishment from seeds alone, and seedlings that flower in their first year of growth (Allcock et al., 2022; Bartenfelder et al., 2022; Jarvis et al., 2012). Because they rely heavily on sexual reproduction, southern edge‐of‐range meadows may represent valuable genetic diversity hotspots among *Z. marina* populations (Diekmann & Serrão, 2012). Importantly, in *Z. marina*, genotypic diversity has been associated with resistance and resilience to natural disturbances (Hughes & Stachowicz, 2004, 2009, 2011; Reusch et al., 2005), and allelic richness and relatedness have both been linked to increased biomass and productivity (Hughes et al., 2016; Stachowicz et al., 2013). Therefore, the mating system can be especially influential in genetic diversity hotspots by impacting standing levels of variation within these populations.

Measures of the genetic mating system include polyandry (i.e., the degree to which females multiply mate), paternity skew (i.e., the extent to which mating can be monopolized by a fraction of the available males), and outcrossing rates (i.e., the proportion of cross‐fertilized versus self‐fertilized progeny). Currently, we have a limited understanding of basic characteristics of the sexual mating system in *Z. marina*. Of the relatively few eelgrass mating system studies, the majority have investigated population‐level patterns in clonal structure and outcrossing rates. Results reveal population‐specific patterns in clone structure (Billingham et al., 2007; Furman et al., 2015; Peterson et al., 2013; Reusch, 2001), related to environmental impacts on life‐history strategies (Harwell & Rhode, 2007). Additionally, there are population‐specific patterns in outcrossing rates in which meadows range from almost entirely outcrossing (Furman et al., 2015; Reusch, 2000b; Ruckelshaus, 1995) to nearly entirely selfing (i.e., self‐fertilizing) in instances of low genotypic diversity (Reusch, 2001). Furthermore, some populations contain spatially organized kin groups (Billingham et al., 2007; Furman et al., 2015; Hays et al., 2021), suggesting that kin interactions have the potential to shape seagrass behavior, life‐history strategy, and resource allocation.

Fine‐scale analyses on individual‐level patterns in mating system have been conducted in perennial meadows and report multiple paternities within inflorescences, confirming that marine angiosperms are polyandrous (Follett et al., 2019; Hays et al., 2021; Reusch, 2000b). Several studies have also shown that within‐meadow hydrodynamics play a significant role in shaping mating systems of *Z. marina* of the Northwestern Atlantic, in which outcrossing rates increase in deep water and near the top of the meadow canopy (Follett et al., 2019, Hays et al., 2021). Importantly, the presence of multiple and varying paternities within a maternal brood (in this case, an inflorescent spathe) can reflect potential differences in male siring success and levels of inbreeding. For example, *Z. marina* genet size in the German Baltic Coast was positively correlated with heterozygosity (Hämmerli & Reusch, 2003a). Similarly, self‐fertilization has been shown to decrease seed set in *Z. marina* (Hämmerli & Reusch, 2003b), suggesting that the impacts of mating system can extend to other plant traits, such as seed size and germination rates, which results in important ecological ramifications for meadow health (Delefosse et al., 2016).

Because edge‐of‐range populations experience annual stress and display a shifting reliance on sexual reproduction (Jarvis et al., 2012), analysis of the mating system as a driver of genetic diversity is needed. Here, we investigate within‐plant patterns in polyandry, siring success and outcrossing in three mixed‐annual eelgrass meadows by genotyping all seeds within a given reproductive shoot. This approach overcomes the limitations of previous work by greatly increasing the sample size per reproductive shoot, enabling an assessment of both structural and temporal patterns of mating system variation. By illuminating the links between life history, sexual reproduction, and genetic variation, this study represents an in‐depth characterization of within‐plant genetic mating system dynamics of *Z. marina* at its southern limit along the western North Atlantic Ocean. More broadly, as eelgrass systems globally have flowering densities and seed outputs that are similar in magnitude despite their differing life‐history strategies (Combs et al., 2021), our results may thus be representative across a wide range of meadow types and locations.

## 2 METHODS

### 2.1 Study organism

During *Z. marina*'s reproductive season, reproductive shoots (i.e., flowering shoots) extend from a basal node and form multiple flowering branches (rhipidia). Each rhipidium contains additional branches with several spathes (i.e., inflorescences) containing flowers (De Cock, 1981; Kuo & Den Hartog, 2006; Thayer et al., 1984). Spathes contain a spadix with rows of both male and female flowers clustered in ratios of 2:1. Flowering occurs in two stages: (1) the flowering and exposure of ovaries and (2) the opening of anthers and release of pollen, each separated by approximately 5 days (De Cock, 1980). Past studies describe sequential flowering of spathes (i.e., temporal dichogamy) within *Z. marina* reproductive shoots beginning with basal structures (De Cock, 1980, 1981). Specifically, one spathe per rhipidium flowers at a time, during which the next spathe of the same rhipidium is not yet entirely developed. The second spathe starts flowering a few days following the first spathe opening its anthers. Temporal dichogamy seemingly does not exist across rhipidia (De Cock, 1980, J. Jarvis, unpubl. data). In the present study, spathe position is therefore used as a proxy for reproductive timing, and rhipidium position is used as a proxy for height within the water column, consistent with previous literature (e.g., Follett et al., 2019; Furman et al., 2015).

### 2.2 Study sites and sample collection

**FIGURE 1:**
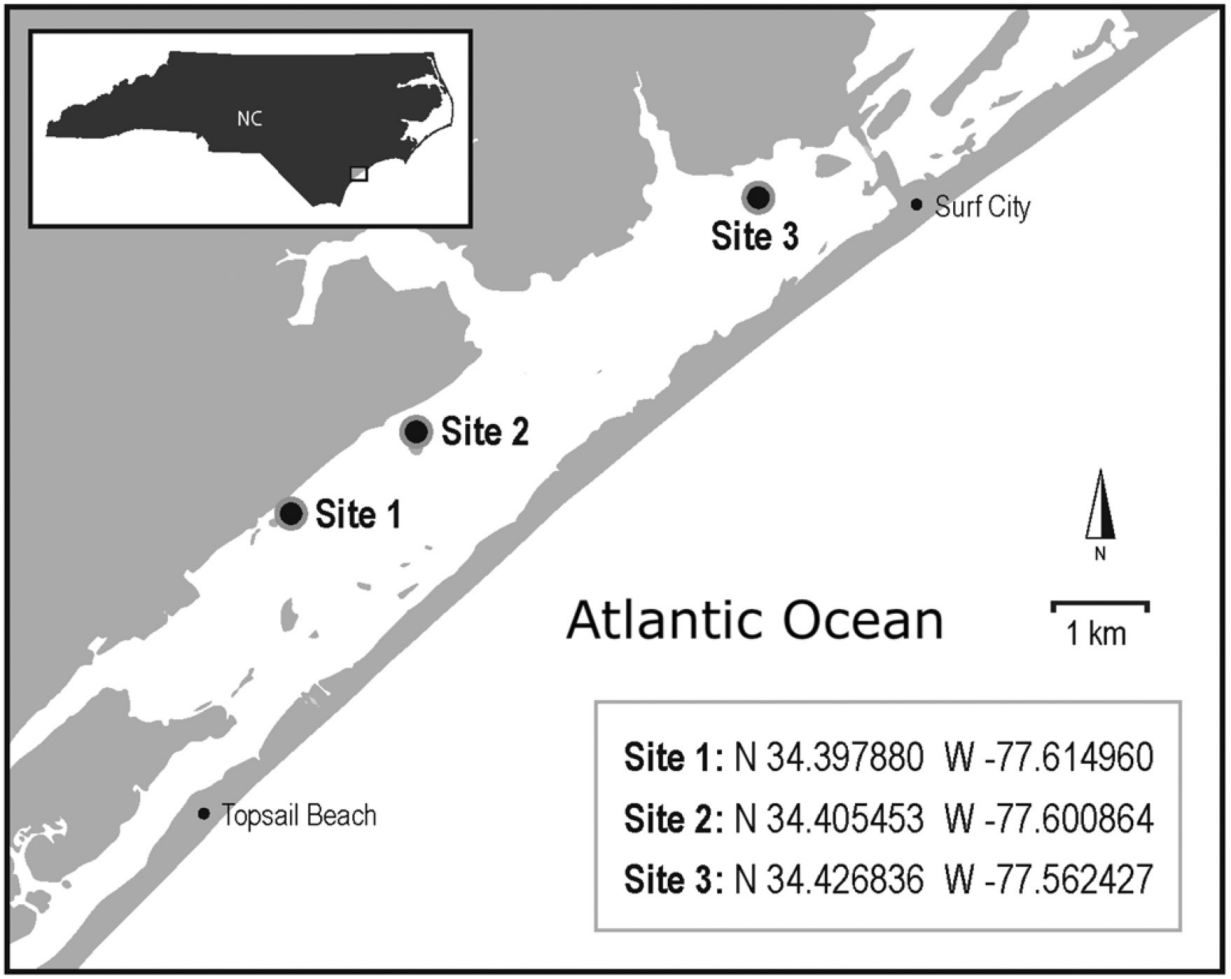
Location of *Zostera marina* meadows sampled within the Topsail Sound Intracoastal Waterway, North Carolina.

**FIGURE 2:**
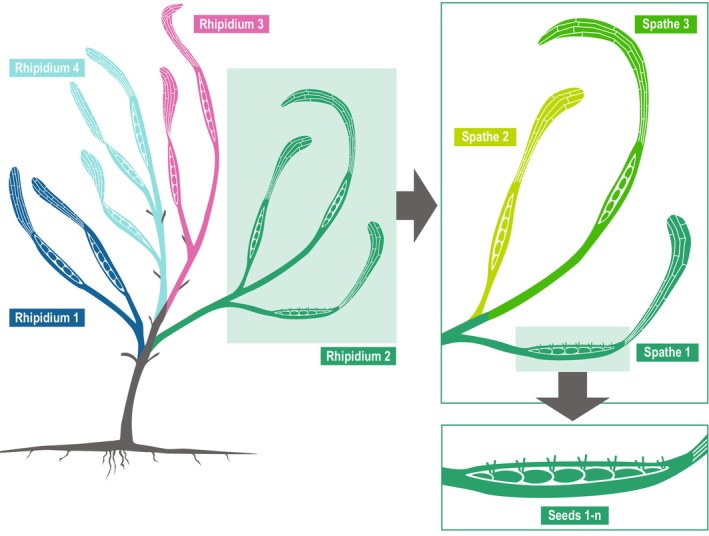
Labeling system of *Zostera marina* flowering shoots. Rhipidia (shown in blue, teal, pink, and green colors) form branching structures on each flowering shoot. Spathes (i.e., inflorescences) of each rhipidium (shown only for Rhipidium 2 in varying shades of green) constitute a branch of a rhipidium and contain a spadix with flowers and eventually seeds.

Three seagrass meadows located in Topsail Sound, North Carolina, were sampled to characterize *Z. marina* mating system variation. The meadows were in a shallow coastal lagoon (34.22 N, 77.37 W) protected from the Atlantic Ocean by Topsail Island (Figure 1). This lagoon contains *Z. marina* in both monospecific and mixed assemblages (i.e., co‐occurring with *Halodule wrightii* and *Ruppia maritima*) (NCDEQ, 2021). Sites were classified as shallow subtidal (depth <2 m MLLW) and were on a narrow shelf between the Intracoastal Waterway and the adjacent shoreline. Twelve flowering shoots were haphazardly collected roughly 5 m apart from each site (*n* = 36 shoots total) on May 4, 2021, and transported on ice to the University of North Carolina Wilmington's Center for Marine Science. Shoots were then cleaned and blotted dry, and the morphological position of each seed was recorded. Each seed was given a position within a spathe, assigned to a given spathe and to a given rhipidium. Labels were assigned in ascending numeric order from increasing proximity to the rhizome (e.g., basal positions were given a value of 1) (Figure 2).

Seeds were removed from spathes, cleaned, blotted dry, and tested for viability using the “squeeze test” by gently compressing individual seeds with a pair of tweezers (Marion & Orth, 2010). Those with a seed coat that compressed were considered nonviable. Viable seeds were removed from the seed coat and genotyped. Historically, the “squeeze test” is performed on seeds that have been released from the flowering shoot; however, if this had been done, the position of the seed within and the identity of the parent plant would not have been known. Moreover, seeds were sampled in May at the peak of the eelgrass reproductive season in North Carolina where most sampled seeds were mature (i.e., at stages 4 and 5; Combs et al., 2021). Indeed, when the sites were re‐visited one‐week post‐sampling, all seeds had been released from their spadices (Jarvis, pers. obs.) indicating that seeds collected as part of this study were mature at the time of collection. Moreover, any immature seeds released from the spadix within 1 week were effectively nonviable; they would not have successfully germinated.

### 2.3 Seed genotyping

DNA was extracted from viable seed samples using a PowerPlant® Pro DNA Isolation Kit. Ten microsatellite loci previously characterized for *Z. marina* (Oetjen et al., 2010; Oetjen & Reusch, 2007; Reusch et al., 1999; Table A1) were amplified in two multiplex Polymerase Chain Reactions (PCR). Individual primer working stocks contained 1 μL of 10 μM fluorescently labeled forward primer and 10 μL each of 50 μM unlabeled forward and reverse primers diluted in 80 μL of ddH_2_O. Primers were then combined into two primer mixes – each containing five different primers (Table A1). PCR conditions for all multiplex conditions were as follows: 95.0°C for 15 min; 2 cycles of 94.0°C for 15 s, 60.0°C for 30 s, 72.0°C for 45 s; 2 cycles of 94.0°C for 15 s, 59.0°C for 30 s, 72.0°C for 45 s; 2 cycles of 94.0°C for 15 s, 58.0°C for 30 s, 72.0°C for 45 s; 2 cycles of 84.0°C for 15 s, 57.0°C for 30 s, 72.0°C for 45 s; 28 cycles of 94.0°C for 15 s, 56.0°C for 30 s, 72.0°C for 45 s; and a final 2 min extension at 72.0°C. Following PCR, two reactions were prepared: one containing 0.5 μL of each PCR product from each of the multiplex mixes. PCR products were added to 9 μL of highly deionized formamide (HiDi) and 0.4 μL of GeneScan‐600 (LIZ) size standard (Applied Biosystems, Foster City, CA, USA) for capillary sequencing on an ABI Prism 3130XL Genetic Analyzer. Fragments were scored using Applied Biosystems Microsatellite Analysis Software (ThermoFisher Scientific Inc.).

### 2.4 Paternity analyses

Known maternal half‐siblings were used for sibship reconstruction and paternity assignment with the maximum‐likelihood approach in COLONY v2.0.7.0 (Jones & Wang, 2010). COLONY parameters included a polygamous mating system for both sexes, inbreeding, and a monecious, diploid species. A long run with medium‐likelihood precision and a genotyping error rate of 1% was performed. Maternal and paternal genotypes were reconstructed using an allele probability threshold of 0.925 for allele calls at each locus. Seeds were categorized as selfed if the putative father had the same genotype as the putative mother. Outcrossing rates were calculated as the proportion of outcrossed offspring per meadow, shoot, rhipidium, and spathe. The effective number of sires and paternity skew per spathe, rhipidium, and shoot were calculated after Neff et al. (2008) in which effective sires = 1/Σ(*rs*_*i*_/seeds)^2^ where *rs*_*i*_ = the number of offspring assigned to sire *i*, and the summation is over all sires contributing to a maternal spathe, rhipidium, or reproductive shoot. Skew was then expressed as 1 – (effective number of sires/actual number of sires). As such, a value of 0 implies no skew in which case all sires contribute equally to a seed set, and a value approaching 1 implies maximal skew in which case nearly all offspring are assigned to a single father. The paternity skew of each sire was calculated as the proportion of genotyped seeds per shoot sired by a particular sire.

Using the reconstructed genotypes, average kinship (*k*), observed and expected heterozygosity (*H*_O_ and *H*_E_, respectively) and clonality were calculated for the parent plants in each meadow using GENODIVE (Meirmans & Van Tienderen, 2004). Following Iacchei et al. (2013), individuals were categorized by levels of kinship (*k*): “nearly identical,” 0.57 > *k* > 0.375; “full siblings,” 0.375 > *k* > 0.1875; “half‐siblings,” 0.1875 > *k* > 0.09375; and “quarter siblings,” 0.09375 > *k* > 0.047 (Loiselle et al., 1995). In addition, the heterozygosity of each paternal genotype was calculated as the proportion of heterozygous loci.

### 2.5 Statistical analyses

Statistical analyses were conducted in RStudio with R v4.2.1 (Posit Team, 2023; R Core Team, 2022), and figures were generated with “ggplot2” and “lattice” (Sarkar, 2008; Wickham et al., 2016). Data were tested for outliers, collinearity, even sample size, and normal distribution (Zuur et al., 2007). To assess patterns in seed viability, a generalized linear mixed model (GLMM) was fit to test the fixed effects of meadow, rhipidium position, and spathe position on the number of viable seeds per spathe (Poisson distribution, offset by the number of seeds per spathe). A random effect of maternal identity was added to account for differences among mothers. To assess patterns in mating system variation, GLMMs with the appropriate distribution were fit to test the fixed effects of meadow, rhipidium position, and spathe position on the response variables of number of outcrossed seeds (Poisson distribution), paternity skew (log + 1 transformed, Gaussian distribution), and number of sires (Poisson distribution) per spathe using the package “lme4” (Bates et al., 2015). A random effect of seeds per spathe was added to control for expected variance due to the fair raffle process in sperm competition (Parker, 1990; Zuur et al., 2007), and random effect of maternal identity was added to account for differences among mothers. Model residuals were visually inspected for normality and homogeneity using the package “DHARMa” (Hartig & Lohse, 2022). Global models were used to perform ANOVAs (*α* = .05) and post hoc pairwise comparisons (*α* = .05) using the package “car” (Fox & Weisberg, 2011).

To explore whether the number of sires was influenced by (a) the number of genotyped seeds, (b) the total number of seeds, and (c) the proportion of selfed offspring, GLMMs (Poisson distribution) were also fit on both a “per spathe” and “per shoot” basis, with a random effect of maternal identity. To assess the impact of paternal genotype on reproductive success, GLMMs (Poisson distribution) were fit to test paternal heterozygosity, whether a sire selfed or outcrossed, and their interaction on (a) the number of seeds sired per male and (b) paternity skew per male, with a random effect of the number of successfully reconstructed loci per sire. Model residuals were visually inspected for normality and homogeneity using the package “DHARMa” (Hartig & Lohse, 2022).

## 3 RESULTS

A total of 1745 seeds were collected from 36 flowering *Z. marina* shoots. On average, there were 3 ± 0.14 rhipidia per shoot (range: 2–5), 3 ± 0.13 spathes per rhipidia (range: 1–9), and 7 ± 0.16 seeds per spathe (range: 1–22). Of the seeds collected, 524 were found to be nonviable. Processing error resulted in the loss of 177 seeds, resulting in 1044 seeds able to be genotyped. During quality control, 198 seeds were removed from the dataset to ensure sufficient replication. Specifically, any rhipidium at position 5; any spathe at position 5, 6, 8, or 9; and any spathe with less than 50% genotyped seeds were removed from analysis. This resulted in a final dataset of 844 seeds from 26 shoots (7 shoots from meadow 1, 11 shoots from meadow 2, 8 shoots from meadow 3). On average, 5 seeds were genotyped per spathe (i.e., 85% of each spathe), 10 seeds per rhipidium (i.e., 85% of each rhipidium), and 29 seeds per shoot (i.e., 84% of each shoot; see Table A2 for additional details on sample sizes per shoot). Among the 524 nonviable seeds, there were, on average, 15 ± 2.3 nonviable seeds per shoot (range: 0–54), 5 ± 0.6 nonviable seeds per rhipidium (range: 0–36), and 2 ± 0.2 nonviable seeds per spathe (range: 0–21).

Overall, 93% of offspring loci were successfully amplified and scored (7849 of 8440 loci). All loci were polymorphic, ranging from two alleles (*ZM10*, meadow 3) to nine alleles (*ZM3*, meadows 1 and 2) (Table A3). Given the small quantity of DNA extracted per seed (~5 ng/μL in a 10 μL volume), re‐runs were rarely possible. The analyses presented below were performed on this complete dataset of 844 seeds. COLONY reconstructed 251 of 260 maternal loci (97%) with an average probability of 0.997 and 1322 of 2080 paternal loci (64%) with an average probability of 0.994. Additional analyses on a reduced data set of 688 seeds that were successfully genotyped at ≥9 loci were also performed and gave qualitatively similar results (see Tables A1, A2, A3, A4, A5 for analyses on the reduced dataset).

### 3.1 Paternity

**FIGURE 3:**
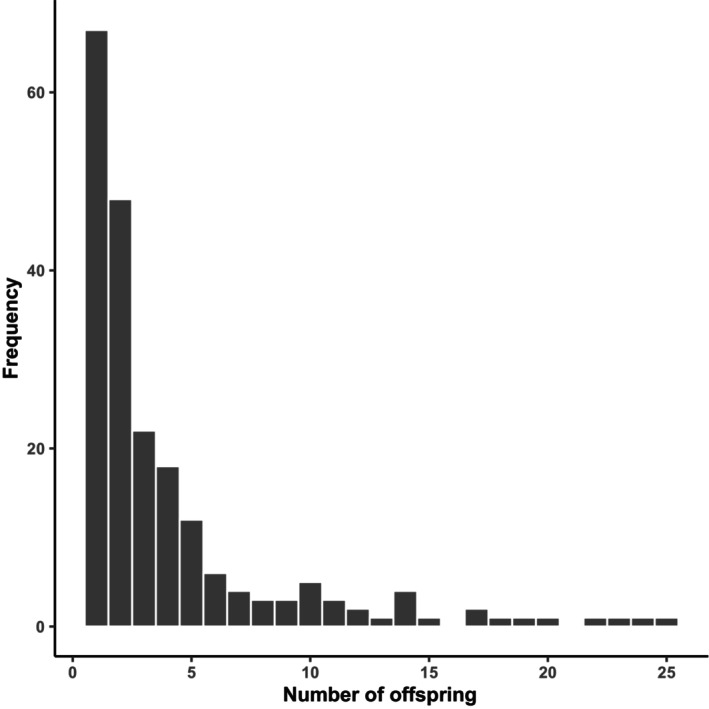
Histogram depicting the frequency of the number of seeds fertilized per sire (*n* = 208 sires).

Multiple sires were present in 100% of shoots, 87% of rhipidia, and 82% of spathes with means of 8 ± 0.6 sires per shoot (range: 3–16), 4 ± 0.3 sires per rhipidium (range: 1–13), and 3 ± 0.1 sires per spathe (range: 1–7) (Table 1). In total, 208 distinct sires were detected, including sires that self‐pollinated. Self‐pollination was detected in 19 out of 26 shoots (73%). No sire was found to have fertilized seeds across meadows or on more than one shoot, though they did fertilize seeds on different rhipidia (2 ± 0.1, range: 1–4) and different spathes (2 ± 0.1, range: 1–12) of the same shoot. A mean of 4 ± 0.3 seeds (range: 1–25) were fertilized per sire, though individual reproductive success varied substantially. Paternity skew was high and varied across spathes (mean = 0.12 ± 0.01, range: 0.00–0.44), rhipidia (mean = 0.23 ± 0.02 range: 0.00–0.56), and shoots (mean = 0.49 ± 0.02, range: 0.23–0.66) (Table 1 **)**, with reported values indicating that most pollen donors fertilized very few seeds, though a small fraction were highly successful (Figure 3). Outcrossing rates per spathe averaged 0.70 ± 0.03, which were similar to outcrossing rates across rhipidia (0.70 ± 0.04), shoots (0.71 ± 0.05), and meadows (0.71 ± 0.01) (Table 1). Among the 26 reconstructed maternal genotypes, no clones were detected, and the average kinship among individuals varied between −0.005 and 0.026, corresponding to kinship values for unrelated individuals (Table 2). Among the 208 reconstructed paternal genotypes, the average kinship among individuals varied between 0.009 and 0.013. Across meadows, observed heterozygosity (*H*_O_) was generally lower than expected heterozygosity (*H*_E_; Table 2).

**TABLE 1:** Mating system characterization of the 26 sampled flowering shoots from meadows in Topsail, NC (*n* = 844 seeds).

Structure	*n*	Outcrossing	*n* sires	*n* fertilized	Skew
Spathe	162	0.70 ± 0.03	3.1 ± 0.1	2.4 ± 0.1	0.12 ± 0.01
Rhipidium	83	0.70 ± 0.04	4.4 ± 0.3	1.8 ± 0.1	0.23 ± 0.02
Shoot	26	0.71 ± 0.05	8.0 ± 0.6	1.0 ± 0.0	0.49 ± 0.02

*Note*: Variables include the number of genotyped morphological structures (*n*), the proportion of outcrossed seeds per genotyped seeds $\bar{x}$ ± SE, the number of sires ($\bar{x}$ ± SE), the number of structures fertilized per sire ($\bar{x}$ ± SE), and paternity skew ($\bar{x}$ ± SE).

**TABLE 2:** Genetic diversity indices of maternal shoots and pollen donors (sires) in three meadows located in Topsail, NC, using genotypes reconstructed from COLONY.

	Meadow 1			Meadow 2			Meadow 3		
	Shoots	Sires	All	Shoots	Sires	All	Shoots	Sires	All
*n*	7	55	62	11	75	86	8	58	66
*k*	0.006	0.013	0.014	−0.005	0.009	0.006	0.026	0.010	0.010
*H*_O_	0.402	0.202	0.240	0.436	0.199	0.247	0.468	0.282	0.318
*H*_E_	0.395	0.329	0.337	0.405	0.335	0.350	0.410	0.380	0.384
*N*_A_	2.7	4.4	4.4	3.4	4.4	4.9	2.7	3.6	3.7

*Note*: Variables include *n* (sample size), *k* (mean kinship coefficient), *H*_O_ (observed heterozygosity), *H*_E_ (expected heterozygosity), and *N*_A_ (number of alleles).

### 3.2 Predictors of mating system variation

**FIGURE 4:**
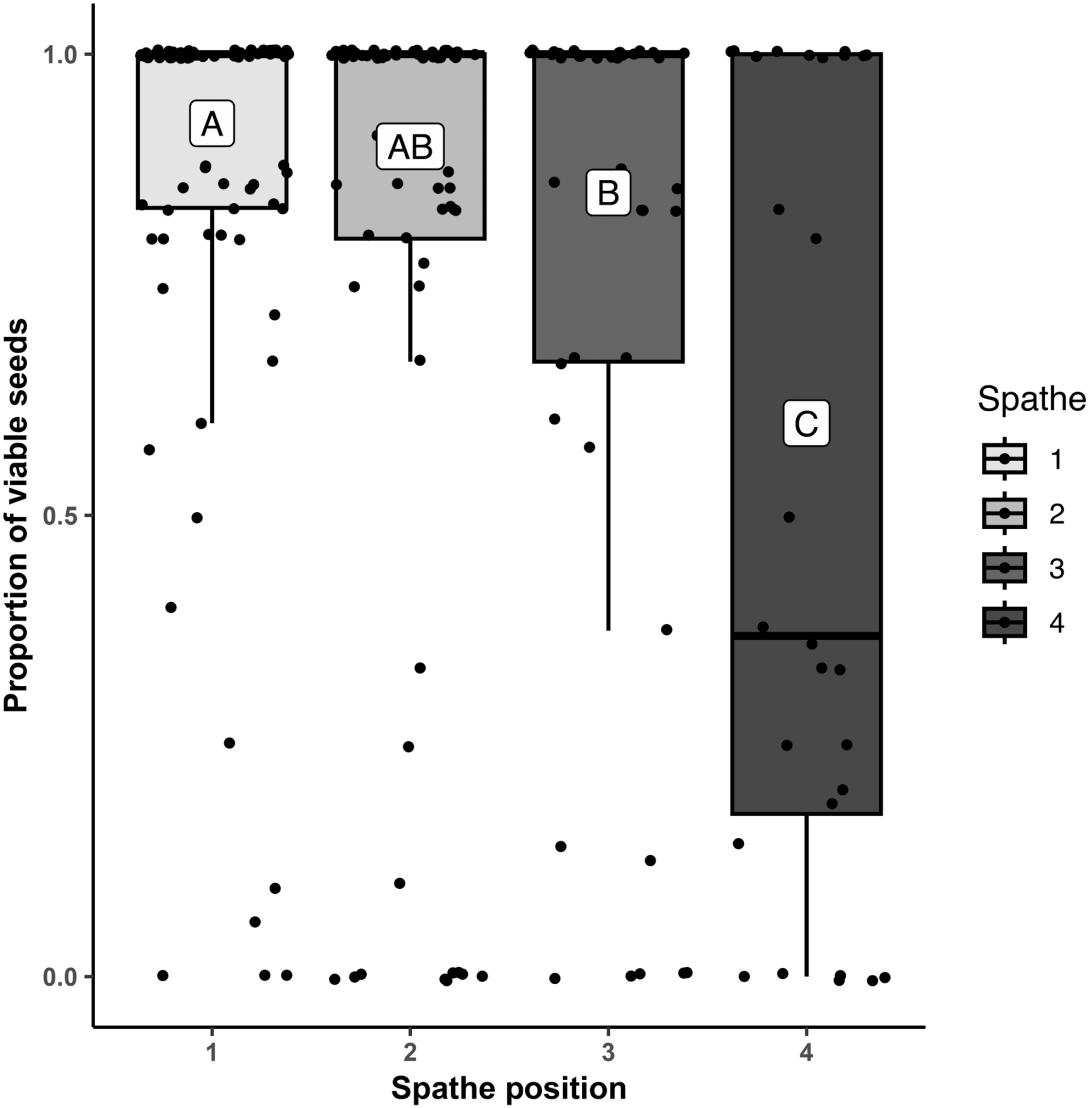
Mean proportion of viable seeds per spathe as a function of spathe position. A, AB, B, and C refer to group bins based on post hoc within‐group pairwise comparisons (*α* = .05). Individual spathe values are plotted using the jitter function in R.

**FIGURE 5:**
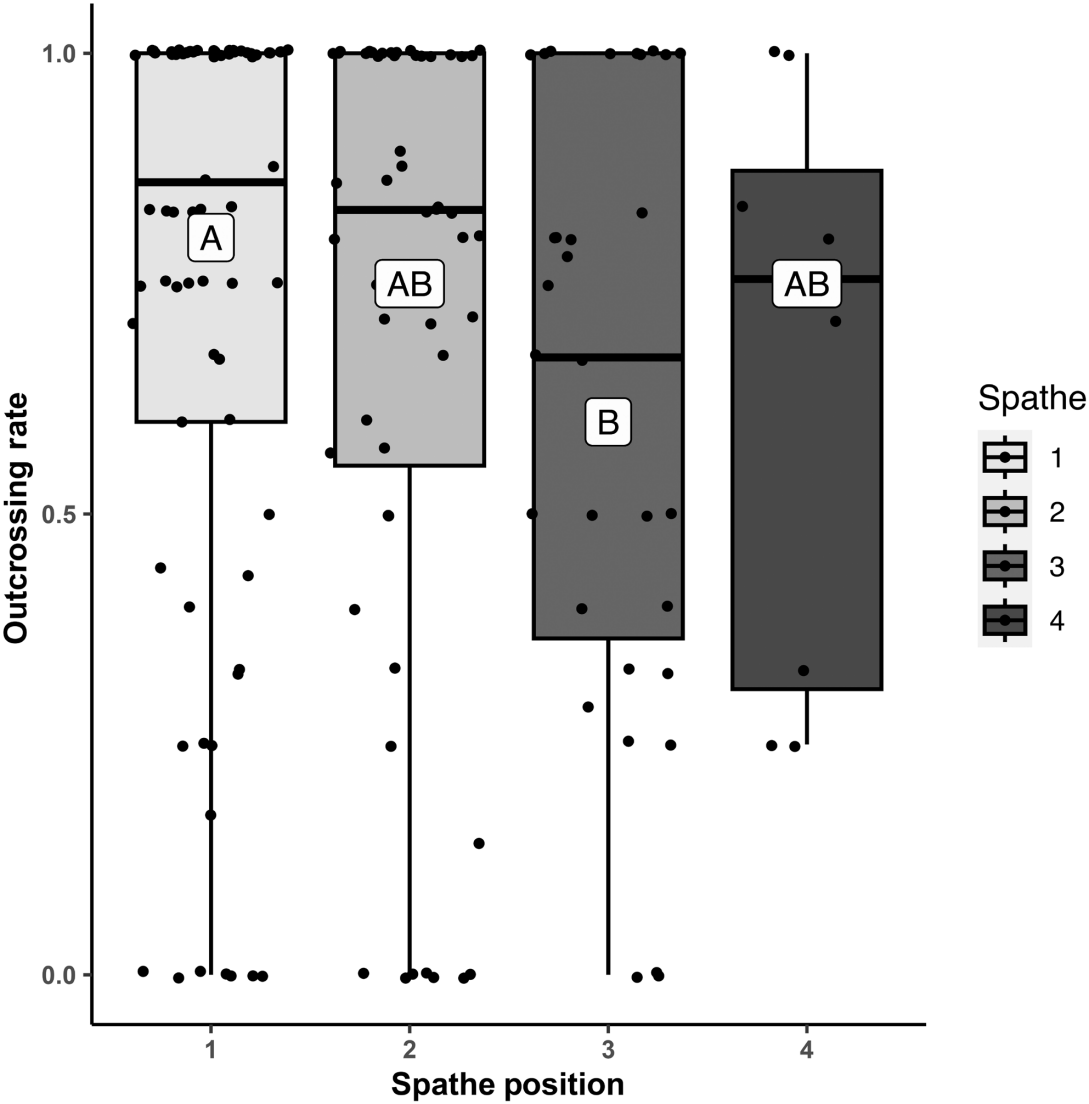
Mean proportion of outcrossed seeds per spathe as a function of spathe position. A, AB, and B refer to group bins based on post hoc within‐group pairwise comparisons (*α* = .05). Individual spathe values are plotted using the jitter function in R.

**FIGURE 6:**
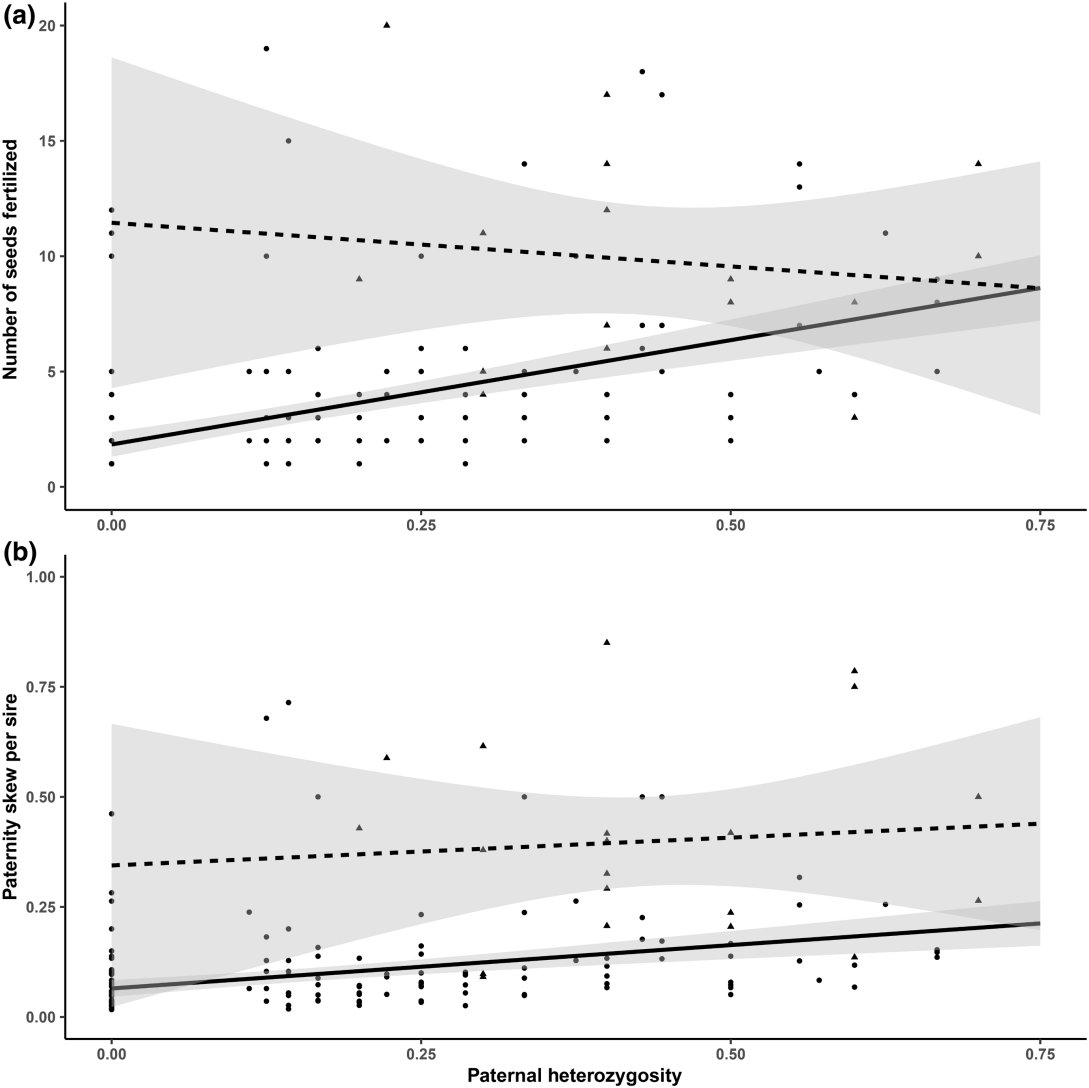
Relationship between paternal heterozygosity and (a) the number of seeds fertilized and (b) paternity skew. Triangles and dashed line correspond to selfing sires; circles and solid line correspond to outcrossing sires.

Meadow and rhipidium position did not significantly influence seed viability, outcrossing rates, number of sires, or paternity skew (Table 3). Similarly, spathe position did not influence number of sires or paternity skew. However, the proportion of viable seeds varied significantly across spathe positions (estimate_spathe 2_ = −0.14 (0.07), estimate_spathe 3_ = −0.25 (0.09), estimate_spathe 4_ = −0.70 (0.12), *χ* ^2^ = 33.5, df_1_ = 3, df_2_ = 215, *p* < .0001; Table 3), decreasing from 0.86 ± 0.03 at spathe position 1 to 0.52 ± 0.07 at spathe position 4 (Figure 4). Outcrossing rates also varied significantly across spathe positions (estimate_spathe 2_ = −0.12 (0.09), estimate_spathe 3_ = −0.38 (0.13), estimate_spathe 4_ = −0.19 (0.22), *χ* ^2^ = 8.77, df_1_ = 3, df_2_ = 158, *p* = .033; Table 3), decreasing from 0.74 ± 0.04 at spathe position 1 to 0.62 ± 0.06 at spathe position 3 (Figure 5). There was a significant, positive correlation between the number of genotyped seeds and the number of sires per spathe (estimate = 0.18 (0.03), df = 159, *p* < .0001; Table 4) and between the total number of seeds and the number of sires per spathe (estimate = 0.15 (0.03), df = 159, *p* < .0001; Table 4). There was a significant, negative correlation between the proportion of selfed seeds and the number of sires per spathe (estimate = −0.76 (0.16), df = 159, *p* < .0001; Table 4). The same patterns were observed on a “per shoot” basis (Table 4). Finally, there was a significant interaction between the paternal traits of heterozygosity and selfing. If a sire outcrossed, the positive effect of heterozygosity on both the number of seeds fertilized (estimate_interaction_ = −1.96 (0.50), df = 204, *p* < .001) and paternity skew (estimate_interaction_ = −1.68 (0.51), df = 204, *p* = .00101) was stronger than if a sire self‐fertilized (Table 5, Figure 6).

**TABLE 3:** Global generalized linear mixed model results for the effects of meadow, rhipidium position, and spathe position on mating system characteristics.

Response	Predictors	df_1_, df_2_	*χ* ^2^	*F*‐ratio	*p*
*n* viable seeds	Meadow	2, 216	1.106	0.205	.575
	Rhipidium	3, 215	6.105	1.512	.107
	Spathe	3, 215	33.499	11.391	**2.53e‐7**
*n* outcrossed seeds	Meadow	2, 159	0.020	0.062	.990
	Rhipidium	3, 158	3.792	1.198	.285
	Spathe	3, 158	8.771	2.973	**.033**
*n* sires	Meadow	2, 159	0.556	3.294	.757
	Rhipidium	3, 158	1.279	0.907	.734
	Spathe	3, 158	4.842	1.322	.184
Paternity skew	Meadow	2, 159	5.715	0.496	.057
	Rhipidium	3, 158	2.495	0.151	.476
	Spathe	3, 158	3.966	3.518	.265

*Note*: Significant predictors (*p* < .05) are indicated in bold.

**TABLE 4:** Generalized linear mixed models results for the effects of the total number of seeds, the number of genotyped seeds, and the proportion of selfed seeds per spathe and per shoot on the number of sires detected.

Response	Predictors	df	Estimate (SE)	*p*
*n* sires/spathe	*n* seeds	159	0.147 (0.029)	**5.81e‐07**
	*n* genotyped seeds	159	0.182 (0.027)	**3.07e‐11**
	Proportion selfed seeds	159	−0.759 (0.162)	**2.66e‐06**
*n* sires/shoot	*n* seeds	23	0.019 (0.005)	**.0001**
	*n* genotyped seeds	23	0.022 (0.005)	**4.56e‐05**
	Proportion selfed seeds	23	−0.335 (0.300)	.265

*Note*: Significant predictors (*p* < .05) are indicated in bold.

**TABLE 5:** Generalized linear mixed models results for the effects of paternal heterozygosity on the number of seeds fertilized and paternity skew.

Response	Predictor	df	Estimate (SE)	*p*
*n* fertilized seeds	Heterozygosity*Selfing	204	−1.9560 (0.5047)	**.000106**
Paternity skew	Heterozygosity*Selfing	204	−1.6775 (0.5103)	**.00101**

*Note*: Significant predictors (*p* < .05) are indicated in bold.

## 4 DISCUSSION

*Zostera marina* meadows growing at the southern edge‐of‐range in the western North Atlantic experience a high degree of thermal stress and annual summer mortality (Bartenfelder et al., 2022; Thayer et al., 1984). As a result, recent shifts from a primarily perennial to a mixed‐annual life‐history strategy have been observed (Jarvis et al., 2012), along with resultant increases in flowering densities (Combs et al., 2021; Jarvis et al., 2012; Thayer et al., 1984) and genetic diversity (Allcock et al., 2022). Our characterization of the mating system in three regional meadows revealed high levels of multiple paternity and outcrossing rates across individual reproductive shoots. Mating systems also varied temporally, with some spathe positions showing significantly higher levels of selfing. Moreover, fertilization was not evenly distributed among individuals as reproductive success varied significantly among males – those with higher heterozygosity and those that self‐fertilized produced greater numbers of seeds and displayed higher reproductive skew (i.e., monopolized a greater fraction of the available seeds). Our findings reveal an edge‐of‐range eelgrass mating system consistent with shifts in life‐history strategy toward greater sexual recruitment and highlight the impact of male traits on reproductive output.

Multiple mating occurred in all sampled shoots and was not significantly different among the three *Z. marina* meadows. The number of sires per spathe (i.e., polyandry) was similar to those found in several other sampled *Z. marina* populations (Follett et al., 2019; Hays et al., 2021), despite the larger number of spathes sampled in this study. Overall, shoots displayed high degrees of polyandry (8 ± 0.6; range: 3–16), which is typical in angiosperms (Pannell & Labouche, 2013), particularly in dense populations (e.g., Friedman & Barrett, 2011). For example, up to nine sires within a single fruit have been reported in the terrestrial angiosperms scarlet gilia (*Ipomopsis aggregata*) (Campbell, 1998), white campion (*Silene latifolia*) (Teixeira & Bernasconi, 2007), and Allegheny monkeyflower (*Mimulus rigens*) (Mitchell et al., 2005). A fair raffle process in sperm competition (Parker, 1990) predicts a positive correlation between the number of seeds and number of sires. That is, larger reproductive shoots are more likely to contain offspring from each of the males a female mates with, simply because of the chance probability that each male's pollen contributes to at least one of the offspring. Indeed, this is reflected in the positive correlation detected between both the number of seeds (total and genotyped) and number of sires. Polyandry was also influenced by the proportion of selfed seeds (Table 4). In instances of high degrees of self‐fertilization, few sires monopolize available ovules, resulting in lower levels of polyandry. Therefore, the observed variance in the number of sires appears to be a function of the fair raffle process and the degree to which the parent plant selfed. In general, polyandrous mating systems may be advantageous as a mechanism for producing genetically diverse progeny (Karron & Marshall, 1990) and selecting for male phenotypic traits that provide the greatest offspring fitness (Lankinen et al., 2009; Schlichting et al., 1990) which, in turn, may lead to population growth (Ashman et al., 2004).

Paternity skew varied markedly within and among flowering shoots and was not correlated with meadow, rhipidium, or spathe position. Some sires fertilized up to 2400% more offspring than others (Figure 3), and such variance in reproductive success is often attributed to variance in either offspring mortality or male quality (Hedgecock, 1994; Hedrick, 2005). Differential patterns of offspring mortality could contribute to paternity skew among males, with some males producing fewer viable seeds than others. As genotyping was only performed on viable seeds that were close to maturity at the time of sampling, the paternal influence on nonviable seeds remains unknown. While there are reviews of hydrophilous (i.e., water‐mediated) pollination in the seagrasses (e.g., Ackerman, 2000; Furman et al., 2015; Ruckelshaus, 1996), there have been no attempts to link pollination to male genotype. Male fertilization success in angiosperms is presumed to be impacted by a variety of factors, including pollen production (Field et al., 2012), pollen clumping (Martin et al., 2009), and pollen competition (Mulcahy & Mulcahy, 1975). Indeed, intraspecific variation in pollen production and viability has been observed in terrestrial angiosperms and linked to male genotype (i.e., some males produce more or better pollen than others), resulting in some males contributing significantly more offspring than others (Danti et al., 2022; Devasirvatham et al., 2012). Additionally, pollen clumping can increase fertilization success in wind‐pollinated species – clumped pollen fertilizes nearby conspecifics, while single pollen fertilizes plants at a greater distance (Martin et al., 2009). Seagrasses are hydrophilous pollinators and the formation of pollen clouds, similar to pollen clumps, has been suggested in *Z. marina* populations from Shinnecock Bay, New York (Furman et al., 2015) and in other seagrass species (McConchie & Knox, 1989). Because sires that self‐fertilized (regardless of heterozygosity) had consistently higher fertilization success than outcrossing males, proximity between anther and stigma also appears to significantly increase males' fertilization success.

While the aforementioned pollen features have not been directly linked to male genotype in *Z. marina*, the positive correlation between male heterozygosity and fertilization success suggests that male quality may play a role in the observed patterns of reproductive variance. Although heterozygosity is not uniformly found to be a reliable proxy for reproductive success (Botero‐Delgadillo et al., 2020; Wetzel et al., 2012), in seagrasses, it has been linked to increased genet size (Hämmerli & Reusch, 2003a). Larger genets, in turn, possess more flowering shoots and thus more pollen, though estimates of clone size and clonal range have not been made in our system.

Outcrossing rates per meadow, shoot, rhipidium, and spathe were high (averaging 70% of genotyped seeds) and similar to other *Z. marina* populations. Within the species' geographic range, reported outcrossing rates are high (Pacific Northwest: 0.66–1.0 (Ruckelshaus, 1995); Pacific Northwest: 0.91 (Ruckelshaus, 1996); German Wadden Sea: 0.96–0.97 (Reusch, 2000b); Western Baltic Sea: 0.70–0.95 (Reusch, 2001); Northwestern Atlantic: 1.0 (Furman et al., 2015); Northwestern Atlantic: 0.88 (Follett et al., 2019); Northwestern Atlantic: 0.79 (Hays et al., 2021)). Approximately, 300 kilometers north of the Topsail, NC sampling sites in the Chesapeake Bay, populations displayed significant heterozygote deficiencies – possibly a consequence of an inbred mating system (Rhode, 2002). The effect of selfing, however, varies by population. When *Zostera* in the western Baltic Sea self‐fertilized, the theoretical fitness of selfed offspring decreased (Reusch, 2001); and in the Ria Formosa, relatedness of parent plants increased rates of seed abortion (Billingham et al., 2007; Zipperle et al., 2011). Alternatively, selfed mating produced more viable seeds in the Chesapeake Bay (Rhode & Duffy, 2004), an area where eelgrass experiences summer heat stress (Hensel et al., 2023) and is recovering as water clarity improves (Lefcheck et al., 2018).

Outcrossing rates did not differ across rhipidium positions, which are often used as proxies for canopy height. In contrast, previous studies in *Z. marina* found that the topmost rhipidium of each shoot displayed high levels of outcrossing, as pollen dispersal was aided by increased water movement at shallower depths within the water column (Follett et al., 2019). Canopy‐mediated hydrodynamics had no observed effect on *Z. marina* mating systems in Topsail, NC, likely due to differences in canopy height and meadow submergence between these meadows and those in northern Massachusetts (Follett et al., 2019). Meadows in northern Massachusetts had a canopy height of 85–135 cm and were submerged in a tidal height of 3 MLLW (Follett et al., 2019); meadows in NC had a canopy height of 5–35 cm and were submerged in a tidal height of 2 MLLW (Jarvis et al., 2022). The shorter canopy height likely reduced the effect of hydrodynamics on mating system variation, and the shallower water depth lessened the effect of above‐canopy water velocity. Hays et al. (2021) also found that meadow depth itself was a significant driver of mating system dynamics. Shallow depth (1 m MLLW) reduced water movement and pollen dispersal, resulting in decreased outcrossing and paternal richness compared to deeper sites (3 and 5 m MLLW). The physical structure of seagrass meadows serves to increase water velocity above the canopy and decrease water velocity within the canopy, and the effect of meadows on water velocity is exaggerated in deeper meadows (Fonseca et al., 1983; Thayer et al., 1984). Therefore, pollen disperses farther when more deeply submerged (Fonseca et al., 1983, Thayer et al., 1984).

Outcrossing rates did differ among spathes, suggesting that mating system characteristics in *Z. marina* can change over time, even within a single plant. Given that selfing appears to increase in younger spathes, within‐shoot phenology may provide a potential explanation for this pattern. When the first spathes open on a given flowering shoot, pollen must come from another ramet. However, as subsequent spathes mature and extend their stigmas, previous spathes are now extending their anthers, allowing for within‐shoot pollen transfer (De Cock, 1980, 1981). Additionally, temporal changes in pollen availability can occur through quantity (e.g., not enough pollen to go around) or quality (e.g., decreased viability, inbreeding depression) limitation (Aizen & Harder, 2007). Pollen quantity limitation has been reported in the Zosteraceae in the Netherlands (Van Tussenbroek et al., 2016) and in the Baltic Sea (Reusch, 2003). The greater proportion of nonviable seeds on younger spathes observed in this study might thus be the result of decreased pollen availability as the season progresses, though viability cannot unequivocally be distinguished from immaturity here as seeds were removed directly from the spathe rather than collected post release.

Seagrass meadows in NC experience frequent natural disturbance from tropical storms (Paerl et al., 2019; Zhang et al., 2022), thermal stress (Bartenfelder et al., 2022; Jarvis et al., 2012), and sedimentation (Mills & Fonseca, 2003), which are known to promote increases in sexual reproduction (Cabaço & Santos, 2012). The high levels of multiple paternity and outcrossing rates are consistent with meadows experiencing high levels of flowering. Moreover, the observed effects of male heterozygosity and temporal shifts in both selfing and viability highlight the contribution of fine‐scale processes to population‐level patterns of mating system variation, genetic diversity, and, ultimately, ecological function (Wimp et al., 2004). Recent literature has emphasized the need to conserve existing meadows before collapse occurs (Van Katwijk et al., 2016) and to prioritize genetically diverse meadows for their enhanced ecosystem services (Reynolds et al., 2012). *Z. marina* edge‐of‐range meadows of the western North Atlantic may represent one such priority, and our findings reveal processes underpinning the genetic composition of this species, which is essential for informed conservation and management efforts.

## AUTHOR CONTRIBUTIONS

**Lauren R. Sgambelluri:** Formal analysis (lead); investigation (equal); methodology (supporting); writing – original draft (lead); writing – review and editing (equal). **Jessie C. Jarvis:** Conceptualization (supporting); methodology (supporting); validation (equal); writing – review and editing (equal). **Stephanie J. Kamel:** Conceptualization (lead); investigation (equal); methodology (lead); validation (equal); writing – review and editing (equal).

## CONFLICT OF INTEREST STATEMENT

The authors declare no conflict of interest.

## Data Availability

All data used in this paper have been made available on DRYAD. Microsatellite forward and reverse primer sequences, parent and offspring microsatellite genotypes, raw and reduced datasets, R code and corresponding .csv files, and data exploration and statistical results have been included. LINK: https://doi.org/10.5061/dryad.dfn2z358x.

## 1

**TABLE A1:** Microsatellite forward and reverse primer sequences.

Primer mix	Microsatellite locus	Dye	Referred to as	Source	Primer sequence (5′–3′)	*n* alleles
A	CL559CONTIG	NED	*ZM1*	Oetjen et al. (2010)	F: CCACTTCCGTAGTTGCTGTT R: CGATGAGGACGATGAGGAAT	7
	CL32CONTIG2	FAM	*ZM2*	Oetjen and Reusch (2007)	F: AATCTGTTGCCACGAAGGAG R: TCACCTTCATCAAGCAGTCG	7
	ZOSMARGA‐3	VIC	*ZM5*	Reusch et al. (1999)	F: CGACGATAATCCATTGTTGTTGC R: GCTTTTCATTTATCCAATAGTTTGC	7
	ZMC12075	PET	*ZM8*	Oetjen and Reusch (2007)	F: CCTCTTTTTTCCTCTCTCTCTCTCT R: CTTCTGCGAATGATGCCATA	8
	ZOSMARCT‐19	FAM	*ZM9*	Reusch (2000a)	F: CCCAAGAAATATAAAATCGGGG R: CTTCTCCTTCCGCCGCTAC	5
B	ZOSMARCT‐3	VIC	*ZM3*	Reusch et al. (1999)	F: TGAAGAAATCCCAGAAATCCC R: AGACCCGTAAAGATACCACCG	10
	CL172CONTIG1	PET	*ZM4*	Oetjen et al. (2010)	F: CTCCTGGACGCAGAAATATG R: GACAAACGTTAATTCAGAAACAAAA	8
	ZOSMARCT‐12	FAM	*ZM6*	Reusch (2000a)	F: CGTTCATCTTGTCCTCGTCC R: TTTCATTTCCATTTCCCACC	4
	ZMC19017	FAM	*ZM7*	Oetjen and Reusch (2007)	F: TCGTCGAGAAAGAGGAGGAA R: TGTTCTGATTCCGTTCTCCA	8
	ZMC19062	NED	*ZM10*	Oetjen et al. (2010)	F: CACTCTCCTCTTTCCGTTCG R: CAGGGGCCTTCCTCTTACTC	4

**TABLE A2:** Sample sizes for each reproductive shoot used in the reported dataset (*n* = 844).

Shoot	Genotyped seeds per shoot (percent)	$\bar{x}$ genotyped seeds per rhipidium (percent)	$\bar{x}$ genotyped seeds per spathe (percent)
1	39 (85)	10 (84)	5 (84)
2	29 (97)	10 (97)	6 (97)
3	21 (81)	7 (82)	5 (79)
4	21 (88)	11 (86)	5 (88)
5	29 (88)	15 (87)	5 (87)
6	26 (90)	7 (89)	5 (91)
7	41 (89)	14 (89)	5 (90)
8	42 (95)	14 (97)	5 (96)
9	37 (86)	9 (91)	5 (88)
10	19 (79)	6 (83)	3 (79)
11	32 (74)	8 (68)	5 (74)
12	38 (79)	13 (80)	6 (78)
13	21 (81)	5 (80)	5 (80)
14	51 (91)	17 (91)	6 (92)
15	34 (81)	11 (81)	5 (82)
16	30 (91)	10 (93)	6 (92)
17	28 (76)	7 (75)	5 (76)
18	20 (87)	5 (86)	5 (86)
19	10 (77)	3 (77)	3 (77)
20	10 (56)	5 (56)	3 (56)
21	33 (66)	17 (66)	6 (66)
22	59 (97)	20 (96)	6 (97)
23	34 (97)	11 (98)	6 (98)
24	57 (80)	14 (81)	5 (81)
25	30 (83)	8 (86)	5 (84)
26	53 (91)	18 (91)	7 (90)

**TABLE A3:** Mating system characterization of original (*n* = 844 seeds) and reduced dataset (*n* = 688 seeds) using only offspring with ≥9 successfully genotyped loci.

	Original dataset	Reduced dataset
*n genotyped*		
Seeds	844	688
Spathes	162	135
Rhipidia	83	76
Shoots	26	26
*Outcrossing rate*		
Per spathe	0.70 ± 0.03	0.76 ± 0.03
Per rhipidium	0.70 ± 0.04	0.78 ± 0.03
Per shoot	0.71 ± 0.05	0.78 ± 0.05
$\bar{x}$ *sires*		
Per spathe	3.1 ± 0.1	2.9 ± 0.1
Per rhipidium	4.4 ± 0.3	4.0 ± 0.3
Per shoot	8.0 ± 0.6	7.1 ± 0.7
$\bar{x}$ *fertilized per sire*		
Spathes	2.4 ± 0.1	2.2 ± 0.1
Rhipidia	1.8 ± 0.1	1.7 ± 0.1
Shoots	1.0 ± 0.0	1.0 ± 0.0
$\bar{x}$ *paternity skew*		
Per spathe	0.12 ± 0.01	0.10 ± 0.01
Per rhipidium	0.23 ± 0.02	0.19 ± 0.02
Per shoot	0.49 ± 0.02	0.43 ± 0.03

**TABLE A4:** Global generalized linear mixed model results for the effects of meadow, rhipidium position, and spathe position on mating system characteristics using the reduced dataset.

Response	Predictors	df	*χ* ^2^	*F*‐ratio	*p*
*n* outcrossed seeds	Meadow	2	3.303	1.137	.192
	Rhipidium	3	6.467	2.022	.091
	Spathe	3	7.774	2.588	**.051**
*n* sires	Meadow	2	0.561	0.512	.755
	Rhipidium	3	3.269	0.994	.352
	Spathe	3	3.147	1.131	.369
Paternity skew	Meadow	2	3.473	1.596	.176
	Rhipidium	3	5.403	1.737	.145
	Spathe	3	1.580	0.527	.664

*Note*: Predictors approaching significance (*p* < .06) are indicated in bold.

**TABLE A5:** Global generalized linear mixed model results for the effects of paternal heterozygosity and selfing on the number of seeds fertilized and paternity skew using various datasets.

Dataset	Response	Predictors	df	Estimate (SE)	*p*
1	*n* fertilized seeds	Heterozygosity	53	3.641 (0.454)	**1.01e‐15**
		Selfed or outcrossed	53	2.073 (0.276)	**5.72e‐14**
		Heterozygosity*Selfing	53	−3.731 (0.642)	**6.18e‐9**
	Paternity skew	Heterozygosity	53	3.261 (0.450)	**4.29e‐13**
		Selfed or outcrossed	53	1.980 (0.277)	**9.25e‐13**
		Heterozygosity*Selfing	53	−3.249 (0.644)	**4.58e‐7**
2	*n* fertilized seeds	Heterozygosity	176	1.731 (0.229)	**3.90e‐14**
		Selfed or outcrossed	176	0.302 (0.332)	.362
		Heterozygosity*Selfing	176	−0.809 (0.572)	.157
	Paternity skew	Heterozygosity	176	1.633 (0.234)	**2.74e‐12**
		Selfed or outcrossed	176	0.388 (0.341)	.254
		Heterozygosity*Selfing	176	−0.752 (0.586)	.199
3	*n* fertilized seeds	Heterozygosity	51	2.119 (0.337)	**3.14e‐10**
		Selfed or outcrossed	51	0.949 (0.282)	**0.0007**
		Heterozygosity*Selfing	51	−1.198 (0.624)	.055
	Paternity skew	Heterozygosity	51	1.794 (0.338)	**1.15e‐7**
		Selfed or outcrossed	51	0.953 (0.287)	**.0008**
		Heterozygosity*Selfing	51	−0.913 (0.635)	.151

*Note*: Significant predictors (*p* < .05) are indicated in bold. Dataset 1 used reconstructed paternal genotypes from the COLONY run with all seeds (*n* = 844); here only sires with an average genotype probability of ≥.925 were used. Datasets 2 and 3 used reconstructed paternal genotypes from the COLONY run with seeds for which ≥9 loci successfully amplified (*n* = 688). Dataset 2 used only paternal loci that were reconstructed with a probability of ≥.925. Dataset 3 used sires with an average genotype probability of ≥.925.
